# *In Vitro* Binding to a Mitochondrial G-Quadruplex-Forming Sequence and Exploratory Acute Tolerability Assessment of a Cyclic Anthraquinone Anticancer Agent

**DOI:** 10.3390/molecules31193397

**Published:** 2026-09-24

**Authors:** Hikaru Fukuda, Yukina Sanada, Satoshi Fujii, Kazuhisa Fujimoto, Masaki Morishita, Naomi Yada, Osamu Takahashi, Manabu Habu, Shigeori Takenaka, Hiroshi Takeuchi, Shinobu Sato

**Affiliations:** 1 Department of Science of Physical Functions, Division of Maxillofacial Surgery, Kyushu Dental University, 2-6-1 Manazuru, Kokurakita-ku, Kitakyushu 803-8580, Fukuoka, Japanr07takahashi@fa.kyu-dent.ac.jp (O.T.);; 2 Department of Applied Chemistry, Kyushu Institute of Technology, 1-1 Sensui-cho, Tobata-ku, Kitakyushu 804-8550, Fukuoka, Japan; 3 Department of Bioscience and Bioinformatics, Kyushu Institute of Technology, 680-4 Kawazu, Iizuka 820-8502, Fukuoka, Japan; sfujii@bio.kyutech.ac.jp; 4 Department of Applied Chemistry and Biochemistry, Kyushu Sangyo University, 2-3-1 Matsukadai, Higashi-ku, Fukuoka 813-8503, Fukuoka, Japan; 5 Department of Comprehensive Dental Practice Education Development, Kyushu Dental University, 2-6-1 Manazuru, Kokurakita-ku, Kitakyushu 803-8580, Fukuoka, Japan; 6 Department of Health Promotion, Division of Oral Pathology, Kyushu Dental University, 2-6-1 Manazuru, Kokurakita-ku, Kitakyushu 803-8580, Fukuoka, Japan; r12yada@fa.kyu-dent.ac.jp; 7 Department of Health Promotion, Division of Applied Pharmacology, Kyushu Dental University, 2-6-1 Manazuru, Kokurakita-ku, Kitakyushu 803-8580, Fukuoka, Japan

**Keywords:** G-quadruplex, mitochondrial DNA, cyclic anthraquinone, cAQ-mBen, *MT-ND3*, acute tolerability

## Abstract

G-quadruplexes (G4s) have attracted considerable attention as molecular targets for the development of anticancer agents with improved selectivity and safety profiles. Cyclic anthraquinone derivatives have been developed as G4-binding ligands, and our previous studies demonstrated that cAQ-mBen preferentially binds to nuclear G4 structures, including telomeric and c-MYC G4s, and exhibits potent anticancer activity. In the present study, we examined the interaction of cAQ-mBen with the mitochondrial G4-forming oligonucleotide mt6363 and performed an exploratory single-dose tolerability assessment in rats. UV–visible absorption titration using the mitochondrial G4-forming sequence mt6363 revealed an association constant (*K*_a_) of 7.5 × 10^5^ M^−1^. CD spectroscopy showed that the overall CD spectral signature of mt6363 remained broadly similar following cAQ-mBen addition. Furthermore, CD melting analysis showed that cAQ-mBen increased the melting temperature of mt6363 by 8.5 °C, demonstrating pronounced stabilization of the mitochondrial G4 structure. These findings demonstrate that cAQ-mBen can bind to and stabilize the mitochondrial DNA-derived G4-forming sequence mt6363 *in vitro*. The animal experiment was designed as a preliminary exploratory acute tolerability assessment (*n* = 3 per group), not as a definitive toxicology study. Following a single intravenous dose of 1000 mg/kg administered at 10 mL/kg, no mortality or overt severe clinical abnormality was observed during the 14-day observation period; however, lower body-weight gain and intake and numerical increases in AST, BUN, and creatinine were observed in the treated animals, and the small sample size precludes statistical demonstration of absence of toxicity. Overall, the present results demonstrate the *in vitro* binding of cAQ-mBen to a mitochondrial DNA-derived G4-forming sequence, while mitochondrial localization, intracellular mtG4 target engagement, sequence selectivity, and definitive safety remain to be established in appropriately designed studies.

## 1. Introduction

G-quadruplexes (G4s) are non-canonical four-stranded nucleic acid structures formed by guanine-rich sequences and have attracted considerable attention as molecular targets for anticancer drug development. G4 structures are enriched in telomeric DNA and the promoter regions of cancer-related genes, including c-MYC and c-KIT, where their stabilization can interfere with telomere maintenance, DNA replication, and transcriptional regulation [1,2,3,4,5]. Accordingly, numerous G4-binding small molecules, including TMPyP4, PIPER, BRACO-19, and pyridostatin, have been developed [6,7,8,9]. Several G4-targeting compounds, such as CX-3543, CX-5461, and QN-302, have progressed to clinical evaluation, supporting G4 structures as pharmacologically relevant targets for anticancer therapy [10,11,12,13].

Importantly, G4 structures are not restricted to the nuclear genome. Human mitochondrial DNA (mtDNA) contains numerous guanine-rich sequences with the potential to form mitochondrial G-quadruplexes (mtG4s) [14,15]. Increasing evidence suggests that mtG4 formation can influence mtDNA replication, transcription, genome stability, and mitochondrial function. Because mitochondria play essential roles in cellular energy metabolism and apoptosis, perturbation of mitochondrial homeostasis through mtG4 stabilization has emerged as a potential strategy for cancer therapy. Indeed, small molecules capable of recognizing or stabilizing mtG4 structures have been reported to alter mitochondrial function and suppress cancer cell proliferation [16]. Thus, mitochondrial G4s represent attractive molecular targets that may contribute to the biological and anticancer activities of G4-binding ligands beyond their established interactions with nuclear G4s.

We have developed cyclic G4-binding ligands based on naphthalene diimide and anthraquinone scaffolds, termed cyclic naphthalene diimides (cNDIs) and cyclic anthraquinones (cAQs), respectively [17,18,19]. These compounds preferentially recognize G4 DNA over duplex DNA and exhibit anticancer activity through interactions with nuclear G4 structures, including human telomeric and c-MYC G4s. In particular, cAQ-mBen showed strong G4-binding activity, weak interaction with duplex DNA, and potent antitumor activity *in vitro* and in vivo with limited apparent adverse effects [19]. Although our previous mechanistic studies primarily focused on nuclear G4 targets, transcriptome analysis of cAQ-mBen-treated cancer cells suggested alterations in mitochondrial function [19]. Notably, the mt6363 G4-forming sequence is located within the mitochondrial *MT-ND3* region, which encodes a subunit of respiratory complex I [14]. These observations prompted us to investigate whether cAQ-mBen can interact with and stabilize this mitochondrial DNA-derived G4-forming sequence. Our previous study established the synthesis, nuclear G4-binding properties, anticancer activity, transcriptomic effects, and tumor-growth inhibition of cAQ-mBen [19]. However, that study did not directly characterize the interaction of cAQ-mBen with a mitochondrial DNA-derived G4-forming sequence and did not include a dedicated single-dose intravenous tolerability assessment in rats. The present study therefore focuses specifically on these two aspects: the *in vitro* interaction of cAQ-mBen with mt6363 and the preliminary characterization of its acute tolerability following high-dose intravenous administration.

In the present study, we investigated the interaction of cAQ-mBen with the mitochondrial G4-forming sequence mt6363 using UV–visible absorption spectroscopy, circular dichroism spectroscopy, CD melting analysis, and exploratory structural modeling (Figure 1). We also performed a preliminary single-dose intravenous tolerability assessment in rats. The principal aim was to characterize the interaction of cAQ-mBen with the mitochondrial DNA-derived G4-forming sequence mt6363 under *in vitro* conditions. The animal experiment was intended to provide descriptive preliminary observations after a single high-dose administration and was not designed as a definitive toxicology study or to establish a maximum tolerated dose.

## 2. Results

### 2.1. Interaction of cAQ-mBen with Mitochondrial G-Quadruplex DNA

To investigate whether cAQ-mBen directly interacts with a mitochondrial G-quadruplex-forming sequence [14], UV–visible absorption titration experiments were performed using the mitochondrial G4-forming sequence mt6363 in 50 mM Tris–HCl buffer (pH 7.4) containing 100 mM KCl at 25 °C. Upon the addition of mt6363 to a solution of cAQ-mBen, marked hypochromicity was observed at 533 nm, corresponding to the anthraquinone chromophore of cAQ-mBen (Figure 2A). The binding affinity of cAQ-mBen for mt6363 was comparable to those previously observed for representative nuclear G4 sequences [19], suggesting that cAQ-mBen recognizes mt6363 as a G4-forming DNA rather than exhibiting demonstrated sequence specificity for mt6363. Because the absorbance change at 533 nm did not reach a clear saturation plateau under the experimental conditions, the titration data were reanalyzed using fixed 1:1 and 2:1 binding models. The fixed 2:1 model provided a substantially better fit to the experimental data than the fixed 1:1 model, as supported by the goodness-of-fit parameters and residual analysis (Figures S1 and S2 and Table S1). Using the fixed 2:1 model, the apparent association constant (*K*_a_) was estimated to be 7.49 × 10^5^ M^−1^. In addition, mole-ratio analysis yielded a breakpoint corresponding to an apparent cAQ-mBen/mt6363 molar ratio of approximately 2.3:1 (Figure S3). Taken together, these analyses support an apparent binding stoichiometry close to 2:1. The preferential interaction of cAQ-mBen with G4 DNA over duplex DNA was demonstrated in our previous study [19], in which cAQ-mBen exhibited only weak binding to a 12-mer duplex oligonucleotide (apparent *K*_a_ ≈ 0.10 × 10^5^ M^−1^). In contrast, the apparent *K*_a_ for mt6363 obtained in the present study was 7.49 × 10^5^ M^−1^. However, a non-G4-forming mutant of mt6363 and unrelated mitochondrial G4 sequences were not examined. Therefore, the present results support preferential binding to the G4-forming mt6363 sequence relative to duplex DNA, but do not establish sequence-specific recognition of mt6363.

To examine whether cAQ-mBen affects the conformation of mt6363, CD spectra were recorded in 50 mM Tris–HCl buffer (pH 7.4) containing 100 mM KCl at 25 °C. mt6363 exhibited a characteristic CD spectrum consistent with G-quadruplex formation under the present experimental conditions (Figure 3). Upon the addition of cAQ-mBen, only slight changes in ellipticity were observed at 266 and 286 nm. Importantly, no substantial change in the characteristic CD profile was observed, indicating that the overall CD spectral signature of mt6363 remained broadly similar following cAQ-mBen addition.

To evaluate the effect of cAQ-mBen on the thermal stability of mt6363, CD melting analysis was performed using 1.5 μM mt6363 in the absence and presence of a threefold molar excess of cAQ-mBen. The CD signals at 265 nm were monitored for both mt6363 alone and mt6363 in the presence of cAQ-mBen. In the absence of cAQ-mBen, mt6363 exhibited a typical melting curve characteristic of G-quadruplex unfolding (Figure 4). Upon the addition of cAQ-mBen, the melting profile was markedly altered, with the ellipticity gradually increasing up to approximately 60 °C, followed by a cooperative melting transition at higher temperatures. Notably, cAQ-mBen increased the melting temperature of mt6363 by 8.5 °C, demonstrating pronounced thermal stabilization of the mitochondrial G4 structure. Together, the UV–visible, CD, and CD melting analyses demonstrate that cAQ-mBen binds to the mitochondrial DNA-derived G4-forming sequence mt6363, while the overall CD spectral signature remains broadly similar following ligand binding, and markedly enhances its thermal stability.

### 2.2. Predicted Binding Mode of cAQ-mBen to mt6363

To explore a possible binding arrangement between cAQ-mBen and mt6363, a computational model of the complex was generated using AlphaFold3. Two cAQ-mBen molecules were explicitly included in the input based on the apparent 2:1 binding model suggested by the UV–visible titration analysis, and potassium ions were also included. The resulting model positioned the two cAQ-mBen molecules near opposite terminal G-quartet faces of mt6363, suggesting possible π–π stacking interactions (Figure 5). Because the ligand stoichiometry was specified in the input, the model does not independently validate the apparent 2:1 stoichiometry. The predicted binding arrangement should therefore be regarded as a structural hypothesis rather than direct evidence of the molecular binding mode.

### 2.3. Reanalysis of Mitochondrial Gene Expression Following cAQ-mBen Treatment

Reanalysis of the RNA-Seq dataset (GSE223310) revealed broad downregulation of multiple mitochondrial DNA-encoded transcripts following cAQ-mBen treatment at 1 and 5 μM for 6 and 24 h (Figure S5 and Table S2). Among these genes, *MT-ND3*, which encompasses the mt6363 G4-forming sequence [14], was significantly downregulated under all four treatment conditions. However, several other mitochondrial transcripts, including *MT-CO3*, *MT-ND4*, *MT-ND4L*, *MT-ND5*, and *MT-CYB*, showed comparable or greater decreases under several conditions. Thus, the observed transcriptional response was not specific to MT-ND3 but rather reflected a broader alteration in mitochondrial gene expression. The location of mt6363 within the *MT-ND3* region therefore provides a possible biological context for the *in vitro* binding results; however, the present data do not establish a specific causal relationship between mt6363 stabilization and *MT-ND3* downregulation.

### 2.4. Exploratory Acute Tolerability Assessment of cAQ-mBen

Rats received a single intravenous administration of cAQ-mBen at 1000 mg/kg using an injection volume of 10 mL/kg, and general clinical observations were performed for 14 days. Body weight, food intake, water intake, and excretion were monitored throughout the observation period (Figure 6). No mortality or overt severe clinical abnormalities were observed during the observation period. Body weight increased in both groups, although weight gain was lower in the cAQ-mBen-treated animals. Food intake, water intake, and excretion were also generally lower in the treated group. Given the small sample size (*n* = 3 per group), these observations are presented descriptively, and the absence of statistically significant differences cannot be interpreted as evidence for absence of toxicity.

Serum biochemical analyses were performed to evaluate potential hepatic and renal effects following the administration of cAQ-mBen (Figure 7). ALT values were similar between the control and cAQ-mBen-treated groups, whereas AST, BUN, and creatinine were numerically higher in the treated animals. Because only three animals were included per group, these findings should be interpreted descriptively and do not exclude treatment-related hepatic, renal, muscular, or systemic effects.

H&E-stained sections of the kidney, liver, and testis were examined by a pathologist (Figure 8). The images shown in Figure 8 are representative fields and were not the sole basis of the histopathological interpretation. No overt inflammatory infiltrates, necrosis, degeneration, or gross structural disruption were identified in the examined sections. However, no formal semiquantitative lesion scoring or morphometric analysis was performed, and the limited number of animals and qualitative nature of the histological assessment do not exclude subtle, focal, or organ-specific toxicity.

Taken together, the animal data provide preliminary descriptive observations following a single high-dose intravenous administration of cAQ-mBen. These findings should not be interpreted as demonstrating the absence of toxicity or as establishing a favorable safety profile.

## 3. Discussion

Our previous study established cAQ-mBen as a G4-targeting anticancer agent with potent antitumor activity both *in vitro* and in vivo [19]. That work primarily focused on representative nuclear G4 targets and anticancer efficacy, while transcriptome analysis revealed the activation of tumor-suppressive pathways, including p53 signaling, apoptosis, and cellular senescence, together with alterations in oxidative phosphorylation-related genes, suggesting the possible involvement of mitochondrial function. However, direct experimental evidence for the interaction of cAQ-mBen with a mitochondrial DNA-derived G4-forming sequence had not previously been obtained. The present study therefore extends our previous findings in two distinct directions: first, by characterizing the *in vitro* interaction of cAQ-mBen with the mitochondrial DNA-derived G4-forming sequence mt6363; and second, by providing preliminary descriptive observations from an exploratory single-dose intravenous tolerability assessment in rats.

UV–visible absorption titration demonstrated the binding of cAQ-mBen to mt6363. Comparison of fixed binding models favored the 2:1 model over the 1:1 model, and independent mole-ratio analysis yielded an apparent cAQ-mBen/mt6363 ratio of approximately 2.3:1. Together, these analyses support an apparent binding stoichiometry close to 2:1, although they do not establish a structurally validated 2:1 binding mode. The spectral changes observed upon mt6363 binding were similar to those previously observed for telomeric and c-MYC G-quadruplexes [19], supporting the ability of cAQ-mBen to bind a mitochondrial DNA-derived G4-forming sequence in addition to its previously characterized nuclear G4 targets. Although previous experiments demonstrated substantially weaker binding of cAQ-mBen to duplex DNA than to G4-forming sequences [19], the present study did not include a G4-disrupting mt6363 mutant or additional mitochondrial G4 sequences. Therefore, the current data support G4-preferential binding but cannot establish sequence-specific recognition of mt6363 or distinguish mt6363 from other G4-forming sequences. Interaction with non-G4-forming G-rich single-stranded DNA was also not directly evaluated and therefore cannot be excluded. CD spectroscopy further showed that the characteristic CD spectral profile of mt6363 remained broadly similar following cAQ-mBen binding. Although this observation is consistent with retention of the overall G4-like spectral features, CD spectroscopy alone does not allow for the assignment or confirmation of a specific G4 topology.

Importantly, CD melting analysis revealed pronounced thermal stabilization of mt6363 by cAQ-mBen, with an increase in melting temperature (Δ*T*m) of 8.5 °C. This degree of stabilization was comparable to that previously observed for telomeric G4 (Δ*T*m = +8.8 °C) [19], indicating that the interaction of cAQ-mBen with mt6363 is sufficiently strong to substantially enhance its thermal stability. Thus, the interaction with mt6363 is not limited to detectable binding but results in substantial stabilization of the mitochondrial G4 structure. Taken together, the UV–visible, CD, and CD melting results provide experimental evidence that cAQ-mBen can bind to and thermally stabilize the mitochondrial G4-forming sequence mt6363, while the overall CD spectral signature remains broadly similar following ligand binding.

The AlphaFold3 model provided a plausible structural arrangement in which two cAQ-mBen molecules were positioned near opposite terminal G-quartet faces of mt6363. However, because two ligand molecules were explicitly supplied in the modeling input, the model does not independently support the apparent 2:1 stoichiometry obtained from the spectroscopic analysis. Although potassium ions were included in the modeling input, the predicted mt6363 G4 structure was not independently validated against an experimentally determined high-resolution structure. Therefore, particularly because the precise G4 topology of mt6363 has not been established experimentally, the AlphaFold3 model should be regarded strictly as a structural hypothesis and not as direct evidence for either the binding stoichiometry or the terminal-stacking arrangement.

The RNA-Seq reanalysis revealed broad downregulation of multiple mitochondrial DNA-encoded transcripts rather than selective repression of *MT-ND3*. Therefore, the transcriptional response is more appropriately interpreted as a broader mitochondrial effect. Although mt6363 is located within the *MT-ND3* region, the present data do not establish that stabilization of mt6363 directly causes *MT-ND3* downregulation. Moreover, because cAQ-mBen has previously been shown to inhibit SAS cell proliferation and to increase Annexin V/PI-positive cells after treatment at 5 μM for 24 h [19], mitochondrial dysfunction and secondary effects of cellular stress or cell death cannot be excluded as contributors to the observed transcriptomic changes. However, the present study does not demonstrate that cAQ-mBen reaches mitochondrial DNA or engages mt6363 in living cells; therefore, the relationship between the *in vitro* mt6363 interaction and the observed transcriptional changes remains hypothetical. Accordingly, the RNA-Seq findings should be interpreted as correlative and hypothesis-generating rather than as evidence of a direct mt6363-dependent transcriptional mechanism. Establishing a causal relationship will require experiments using a G4-disrupting mt6363 mutant or another appropriate non-G4 control.

Because cAQ-mBen demonstrated promising antitumor activity in tumor-bearing mice in our previous study [19], further characterization of its tolerability is relevant to its preclinical evaluation [20]. In the present study, we performed an exploratory single-dose intravenous tolerability assessment in rats using cAQ-mBen at 1000 mg/kg. This dose was selected as an exploratory high-dose level with reference to previous animal studies of anticancer agents and was not established as a maximum tolerated dose (MTD) or maximum feasible dose (MFD) by a formal dose-escalation study. The regimen was intended to probe acute tolerability and should not be interpreted as representing a clinically relevant exposure; pharmacokinetic data linking this dose to human exposure are not available. No mortality or overt severe clinical abnormalities were observed during the 14-day observation period. However, body-weight gain, food intake, water intake, and excretion were generally lower in the cAQ-mBen-treated animals, and AST, BUN, and creatinine were numerically higher than those in the control group. Because only three animals were included per group, the study was not adequately powered for inferential conclusions regarding the presence or absence of toxicity. Accordingly, these findings are interpreted descriptively, and failure to reach statistical significance is not taken as evidence of safety. Histopathological examination of H&E-stained kidney, liver, and testis sections did not reveal overt inflammatory infiltration, necrosis, degeneration, or gross structural disruption. The displayed micrographs represent selected fields, whereas the histopathological interpretation was based on the microscopic examination of sections from each animal by a pathologist. Nevertheless, no formal semiquantitative lesion scoring or morphometric analysis was performed, and subtle, focal, or organ-specific changes cannot be excluded. A definitive toxicological characterization will require larger group sizes, multiple dose levels, prospectively defined humane endpoints, formal dose-escalation studies, repeated-dose administration, pharmacokinetic analysis, and more systematic quantitative histopathological assessment.

Several limitations should be acknowledged. First, the interaction between cAQ-mBen and mitochondrial G-quadruplexes was demonstrated using *in vitro* DNA models, and further studies employing mitochondrial colocalization imaging, mitochondrial fractionation, mitochondrial G4 imaging, or other cellular target-engagement approaches will be required to determine whether cAQ-mBen reaches mitochondria and interacts with mtG4 structures in living cells. Second, the animal experiment was limited to a single high-dose administration with a small number of animals and should therefore be regarded as an exploratory tolerability assessment rather than a definitive toxicology study.

Overall, the present study expands our previous findings by providing *in vitro* characterization of the interaction between cAQ-mBen and the mitochondrial DNA-derived G4-forming sequence mt6363, together with preliminary exploratory tolerability observations in rats. The animal data should be viewed as hypothesis-generating and as a basis for the design of adequately powered, dose-ranging toxicology studies rather than as evidence that cAQ-mBen is safe.

## 4. Materials and Methods

### 4.1. Materials

DNA oligonucleotide mt6363 (5′-AGGGACGCGGGCGGGGGATATAGGGT-3′, chrM:10182-10207) was purchased from Hokkaido System Science (Sapporo, Japan). Before use, mt6363 was annealed in 50 mM Tris–HCl buffer (pH 7.4) containing 100 mM KCl by heating at 95 °C for 10 min and cooling to 25 °C at 0.5 °C/min. All DNA concentrations reported in this study refer to oligonucleotide strand concentrations. For the intramolecular G4-forming sequence mt6363, one strand corresponds to one potential folded G4 unit. All other chemicals were purchased from Fujifilm Wako Pure Chemical Corporation (Tokyo, Japan), Kanto Chemical (Tokyo, Japan), Tokyo Chemical Industry (Tokyo, Japan), and Sigma-Aldrich (St. Louis, MO, USA). cAQ-mBen was synthesized according to our previously reported procedure [19].

### 4.2. Absorption Titration of cAQ-mBen with mt6363

UV–visible absorption spectra were recorded at 25 °C using a JASCO V-750 spectrophotometer (Tokyo, Japan) and a quartz cuvette with a 1-cm path length. Titration experiments were performed by the incremental addition of mt6363 to a solution of cAQ-mBen (10 μM) in 50 mM Tris-HCl buffer (pH 7.4) and 100 mM KCl. After each addition of mt6363, the solution was mixed and allowed to equilibrate for 1 min before spectral acquisition. UV–visible spectra were recorded up to 800 nm to monitor possible turbidity or light-scattering effects associated with aggregation. No appreciable increase in long-wavelength absorbance or baseline elevation was observed during the titration, and no visible precipitation or turbidity was detected. Absorbance changes at 533 nm were normalized as 1 − *A*/*A*_0_, where *A*_0_ and *A* represent the absorbance in the absence and presence of mt6363, respectively. The normalized absorbance changes were analyzed using the following binding isothermal model [21] in Equation (1).

(1) $$1-\frac{A}{A_{0}}=\frac{1}{2}R_{b}\left\{\frac{1}{K_{a}}+L_{T}+nS_{T}-\sqrt{{\left(\frac{1}{K_{a}}+L_{T}+nS_{T}\right)}^{2}-4nS_{T}L_{T}\;}\right\}$$

where *L*_T_ is the total ligand concentration, *S*_T_ is the total DNA concentration, *n* is the binding stoichiometry, and *R*_b_ has units of concentration^−1^.

To avoid the simultaneous fitting of *K*_a_, *n*, and *R*_b_, the titration data were analyzed using fixed 1:1 (*n* = 1) and 2:1 (*n* = 2) ligand/DNA binding models. The goodness-of-fit of the two models was compared using R^2^, adjusted R^2^, residual sum of squares (RSS), Akaike information criterion (AIC), Bayesian information criterion (BIC), and residual analysis. The apparent association constant (*K*_a_), its standard error (SE), and 95% confidence interval were obtained from the fixed 2:1 model.

Binding stoichiometry was also evaluated independently using the mole-ratio method. The breakpoint was determined as the intersection of linear regressions fitted separately to the low- and high-mt6363-concentration regions.

### 4.3. CD Spectra Measurements of DNA upon Addition of cAQ-mBen

CD spectra were recorded at 25 °C using a JASCO J-820 circular dichroism spectrometer (Tokyo, Japan) and a 1-cm path-length quartz cell. A DNA sample (typically 1.5 μM) in 50 mM Tris-HCl buffer (pH 7.4) and 100 mM KCl was measured in the absence and presence of cAQ-mBen. Thermal melting experiments were performed by monitoring the CD signal at the characteristic wavelength for G4 structures while increasing the temperature at a constant rate.

### 4.4. Tm Measurements

CD melting experiments were performed using 1.5 µM DNA in 50 mM Tris–HCl buffer (pH 7.4) containing 100 mM KCl in the absence or presence of cAQ-mBen. The CD signal at 265 nm was monitored for both mt6363 alone and mt6363 in the presence of cAQ-mBen. The temperature was increased from 20 to 95 °C at a rate of 1 °C/min. The melting temperature (Tm) for each condition was determined using the midpoint method.

### 4.5. AlphaFold3 Structural Modeling

Structural modeling of the cAQ-mBen–mt6363 complex was performed using AlphaFold3 [22]. Two cAQ-mBen molecules were included in the input based on the apparent 2:1 binding model obtained from the UV–visible titration analysis. Potassium ions were also included in the modeling input. The predicted mt6363 G4 structure was not constrained to, or independently validated against, an experimentally determined high-resolution structure. Accordingly, the resulting model was used only to explore a plausible ligand-binding arrangement and not to determine binding stoichiometry or establish the actual binding pose.

### 4.6. Reanalysis of RNA-Seq Data

RNA-Seq data generated in our previous study are publicly available in the Gene Expression Omnibus (GEO) under accession number GSE223310. Briefly, SAS cells were treated with cAQ-mBen at 1 or 5 μM for 6 or 24 h. Two independent biological replicates derived from independently cultured SAS cells were analyzed for each treatment condition. Differential gene expression analysis was performed using edgeR as previously described [19]. Read counts were normalized using the trimmed mean of M-values (TMM) method implemented in edgeR and expressed as counts per million (CPM) and logCPM values. In the present study, the dataset was reanalyzed with a focus on mitochondrial DNA-encoded genes. Genes with a false discovery rate (FDR) < 0.05 were considered significantly differentially expressed. The log2 fold changes of mitochondrial DNA-encoded genes under each treatment condition were visualized as a heatmap.

### 4.7. Animals and Housing

Male Sprague-Dawley rats (5 weeks old, weighing approximately 150 g; Slc:SD, Japan SLC, Inc., Hamamatsu, Japan) were used. The animals were acclimated for one week prior to the experiments and maintained under specific pathogen-free (SPF) conditions. Housing was provided in a temperature-controlled environment with a 12-h light/dark cycle, with ad libitum access to standard laboratory chow and water. All experimental protocols were approved by the Animal Care and Use Committee of Kyushu Dental University (Approval No. [24-028]) and conducted in accordance with the institutional guidelines for animal welfare.

### 4.8. Experimental Design and Administration

Rats were randomly assigned to two groups (*n* = 3 per group): the control group and the cAQ-mBen-treated group. cAQ-mBen was converted to its hydrochloride form prior to administration. Briefly, 170 mg of cAQ-mBen was dissolved in approximately 300 μL of methanol, followed by the addition of 20 μL of 35% hydrochloric acid. The solvent was removed under a stream of nitrogen gas, and the residue was dissolved in approximately 1 mL of water. The solution was subsequently diluted with physiological saline to a nominal final concentration of 100 mg/mL. cAQ-mBen was then administered as a single slow intravenous injection via the tail vein at a dose of 1000 mg/kg using an injection volume of 10 mL/kg. Control animals received an equivalent volume of physiological saline. The 1000 mg/kg dose was selected as an exploratory high-dose level with reference to previously reported acute toxicity and safety studies of anticancer agents and was not established as a maximum tolerated dose (MTD) or maximum feasible dose (MFD) by a formal dose-escalation study. All intravenous administrations were performed under isoflurane inhalation anesthesia to minimize procedural distress.

### 4.9. Clinical Observations

Following administration, rats were monitored daily for 14 days for changes in physical appearance, behavior, and activity. Body weight, food and water consumption, and excreta output were recorded daily. The approved animal protocol classified the procedures as involving mild or transient stress/pain and specified isoflurane anesthesia for the intravenous procedure and isoflurane overdose for euthanasia at the end of the observation period. No numerical humane-endpoint or dose-interruption thresholds were prospectively specified in the approved exploratory protocol.

### 4.10. Serum Biochemical Analysis

On day 14, blood samples were collected under deep isoflurane anesthesia. To obtain serum, the samples were allowed to clot at room temperature for 60 min and then centrifuged at 2800 rpm for 20 min at 4 °C. The supernatant was harvested and stored at −80 °C until analysis. The following biochemical markers were quantified using an automated analyzer: alanine aminotransferase (ALT), aspartate aminotransferase (AST), blood urea nitrogen (BUN), and creatinine (CRE).

### 4.11. Histopathological Examination

After blood collection, animals were euthanized by an overdose of isoflurane. The kidney, liver, and testis were harvested, fixed in 10% neutral buffered formalin, and embedded in paraffin. H&E-stained sections were examined by a pathologist under light microscopy for overt abnormalities such as inflammatory infiltrates, degeneration, necrosis, and structural disruption. The fields shown in Figure 8 were selected as representative images and were not the sole basis of interpretation. No formal semiquantitative lesion score or morphometric analysis was performed.

### 4.12. Statistical Analysis

Statistical analyses were performed for exploratory comparisons between the control and cAQ-mBen groups. Data are presented as the mean ± SD. For longitudinal outcomes (body weight, water intake, food intake, and excretion amount), each animal was summarized by AUC (days 1–14) and by the day 14 value. Blood biochemical parameters (ALT, AST, BUN, and CRE) were analyzed as end-point variables.

Because the animal experiment included only three animals per group and was intended as an exploratory tolerability assessment, animal data were summarized primarily descriptively. Data are presented as the mean ± SD, with individual values shown where applicable. Longitudinal outcomes and endpoint serum-biochemistry values were not interpreted on the basis of failure to reach a conventional significance threshold.

With *n* = 3 versus *n* = 3, exact two-sided rank-based or permutation tests have insufficient resolution to establish statistical significance at the conventional 0.05 level even under complete group separation. Therefore, inferential *p* values were not used to support claims of absence of toxicity.

No claim of equivalence, non-inferiority, or absence of toxicity was made from these exploratory data.

## 5. Conclusions

In conclusion, this study demonstrates that cAQ-mBen binds to and thermally stabilizes the mitochondrial DNA-derived G4-forming sequence mt6363 under *in vitro* conditions, while the overall CD spectral signature remains broadly similar following ligand binding. These findings support an *in vitro* interaction between cAQ-mBen and mt6363 but do not establish mitochondrial localization or intracellular target engagement. The AlphaFold3 model provides a structural hypothesis for a possible binding arrangement, whereas the RNA-Seq reanalysis provides correlative, hypothesis-generating observations regarding mitochondrial gene expression. The exploratory rat experiment provided preliminary descriptive observations following a single intravenous dose of 1000 mg/kg administered at 10 mL/kg; however, the small sample size and limited toxicological evaluation preclude definitive conclusions regarding safety. Further cellular validation and adequately powered toxicological studies will be required to determine the biological relevance and preclinical tolerability of cAQ-mBen.

## Figures and Tables

**Figure 1:**
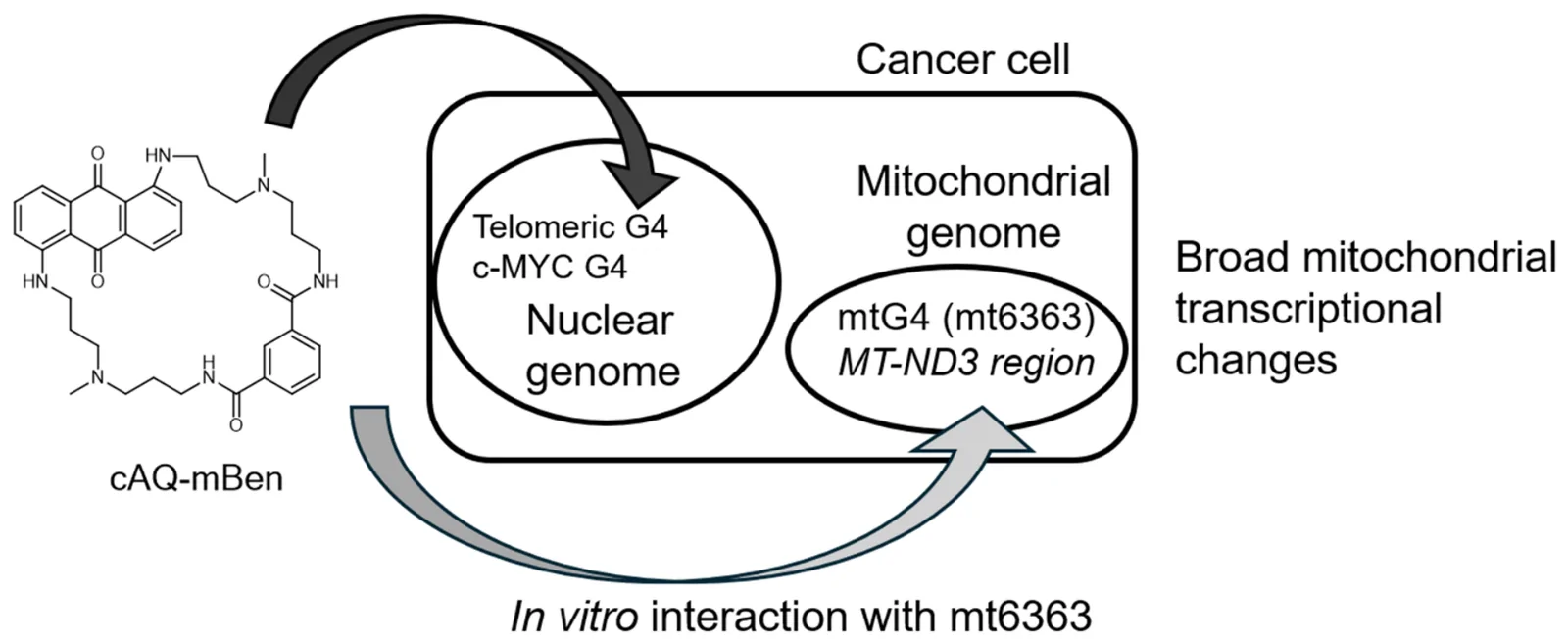
Schematic representation of the proposed nuclear and mitochondrial G-quadruplex targets of cAQ-mBen. cAQ-mBen has previously been shown to recognize nuclear G4 structures, including telomeric and c-MYC G4s [19]. In the present study, the mitochondrial G4-forming sequence mt6363, located within the *MT-ND3* region, was investigated as an *in vitro* binding substrate for cAQ-mBen. Reanalysis of the RNA-Seq data revealed broad mitochondrial transcriptional changes, including the downregulation of *MT-ND3* following cAQ-mBen treatment.

**Figure 2:**
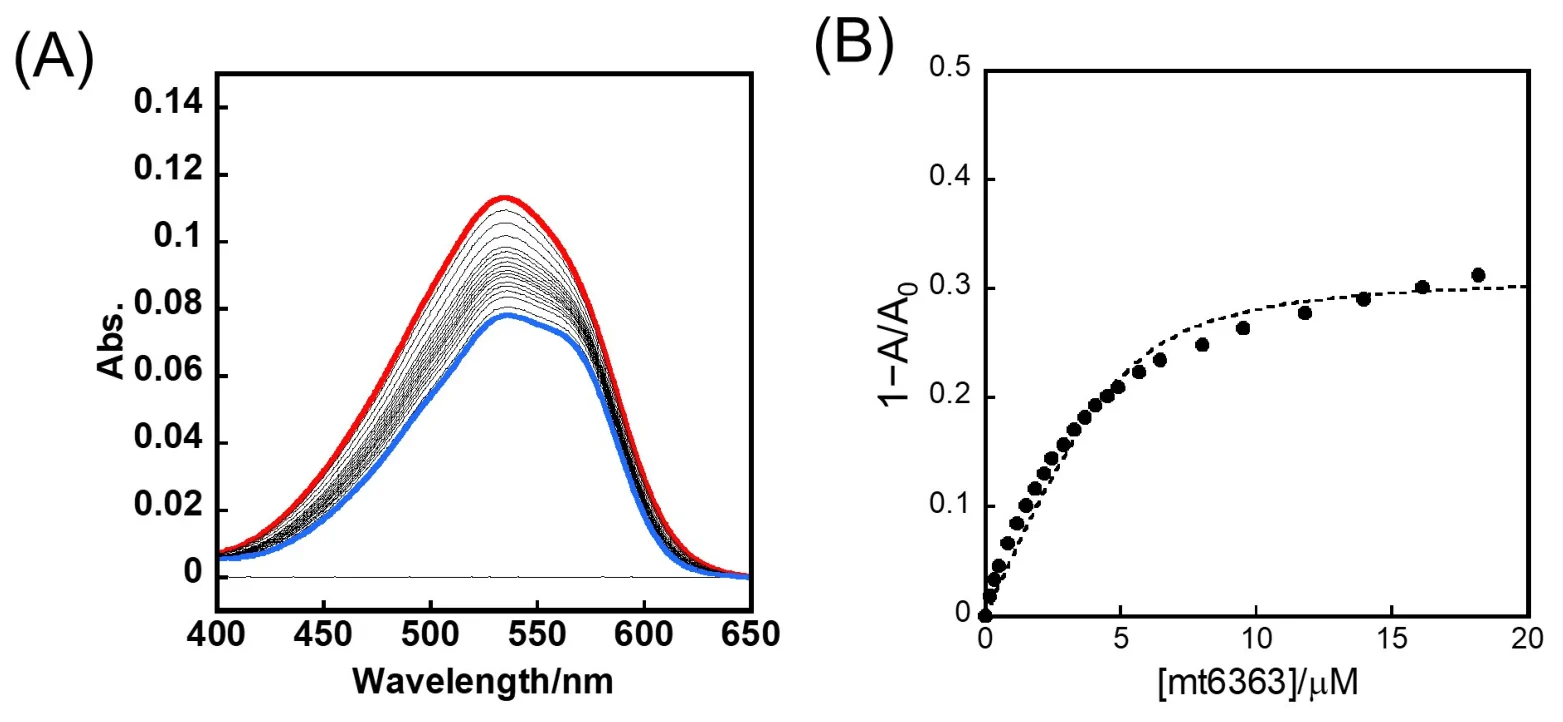
UV–visible absorption titration analysis of the interaction between cAQ-mBen and mt6363. (**A**) UV–visible absorption spectra of cAQ-mBen (10.0 μM) upon the incremental addition of mt6363. The red spectrum represents cAQ-mBen alone, and the blue spectrum represents cAQ-mBen in the presence of 18.2 μM mt6363. Intermediate spectra correspond to intermediate mt6363 concentrations. (**B**) Binding isotherm obtained from the absorbance changes at 533 nm. The filled circles represent the experimental data, and the dashed line represents the fitted curve obtained using the fixed 2:1 binding model. Measurements were performed in 50 mM Tris–HCl buffer (pH 7.4) containing 100 mM KCl at 25 °C. Model comparison and stoichiometric analyses are provided in Figures S1–S3 and Table S1.

**Figure 3:**
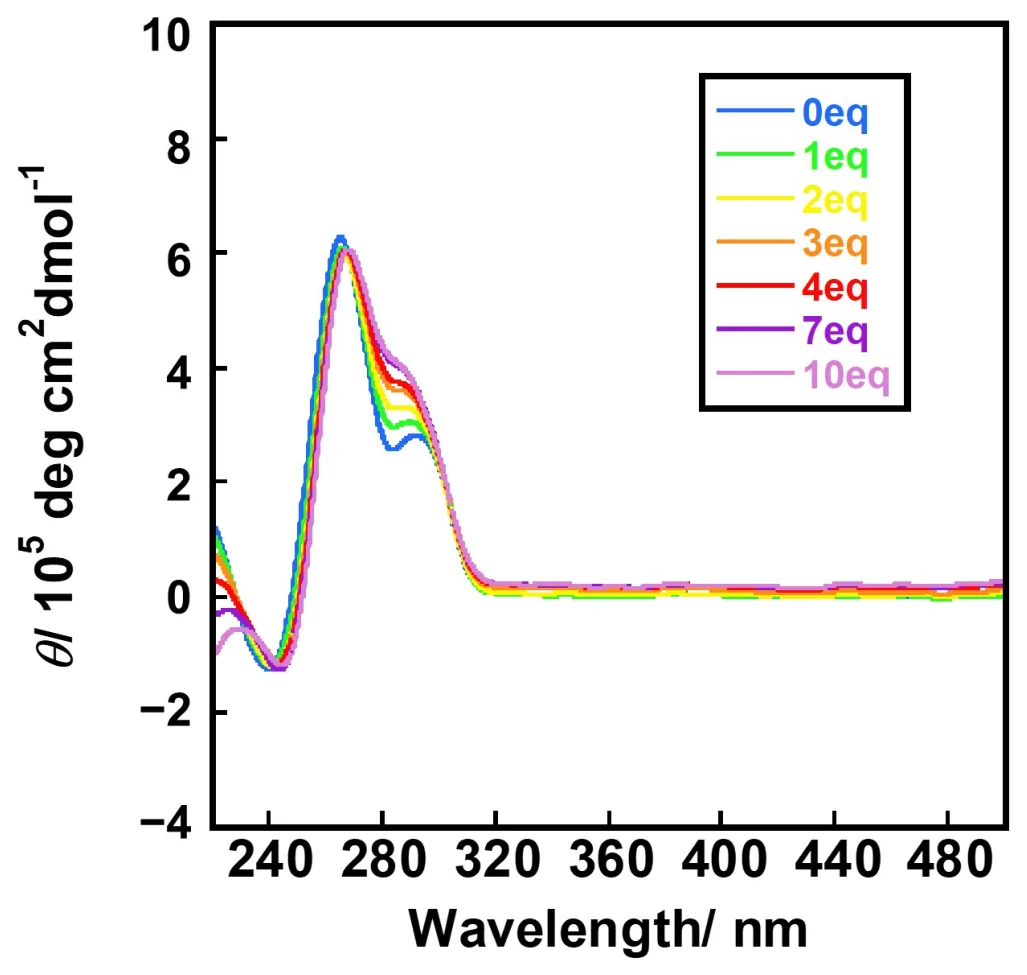
Effect of cAQ-mBen on the CD spectrum of the mitochondrial G-quadruplex-forming sequence mt6363. CD spectra of mt6363 (1.5 μM) in the presence of 0, 1, 2, 3, 4, 7, and 10 molar equivalents of cAQ-mBen. Measurements were performed in 50 mM Tris–HCl buffer (pH 7.4) containing 100 mM KCl at 25 °C.

**Figure 4:**
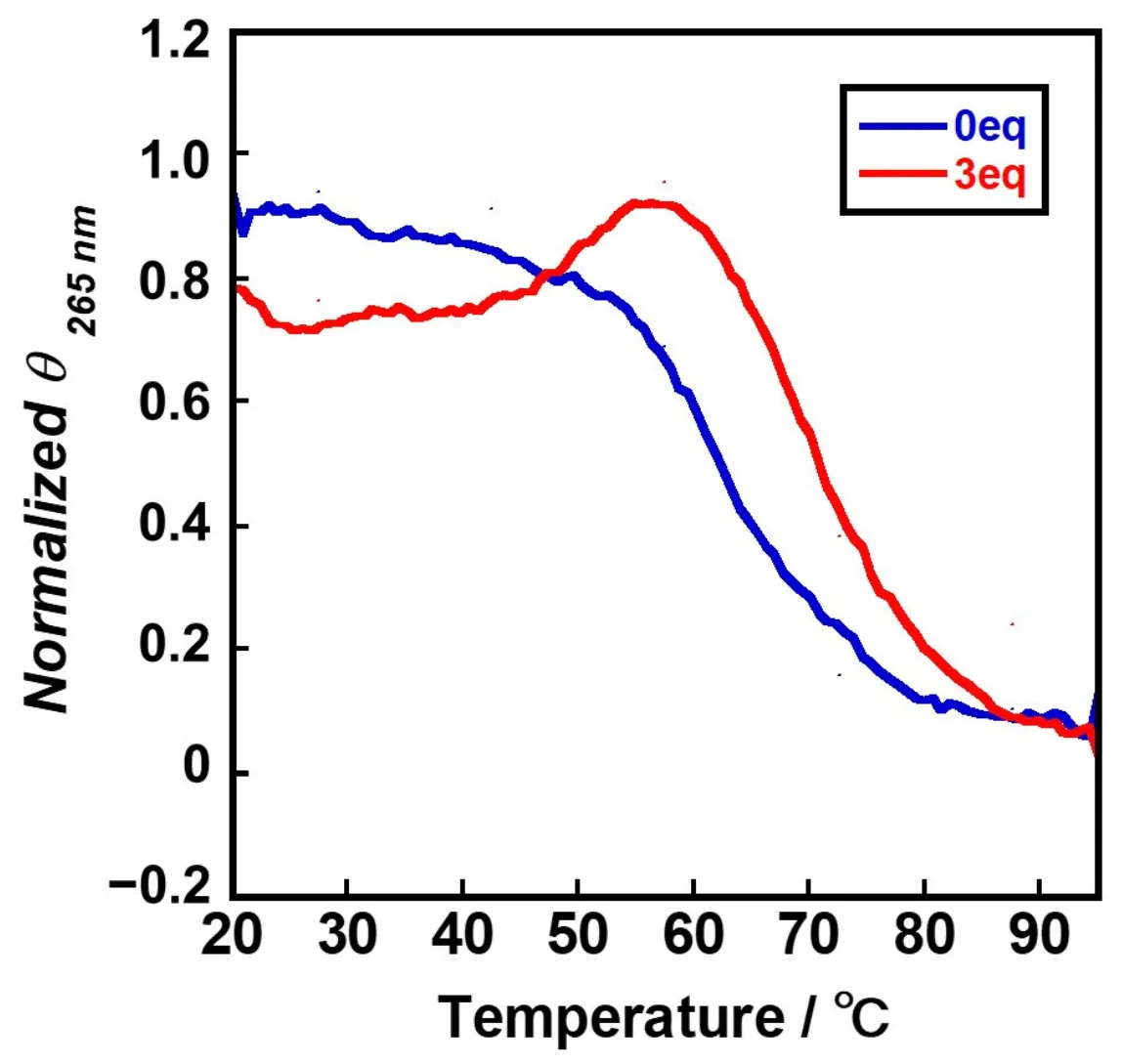
Thermal stabilization of the mitochondrial G-quadruplex mt6363 by cAQ-mBen. CD melting profiles of mt6363 (1.5 μM) in the absence (blue) and presence (red) of a threefold molar excess of cAQ-mBen in 50 mM Tris–HCl buffer (pH 7.4) containing 100 mM KCl. CD signals were monitored at 265 nm for both mt6363 alone and in the presence of cAQ-mBen.

**Figure 5:**
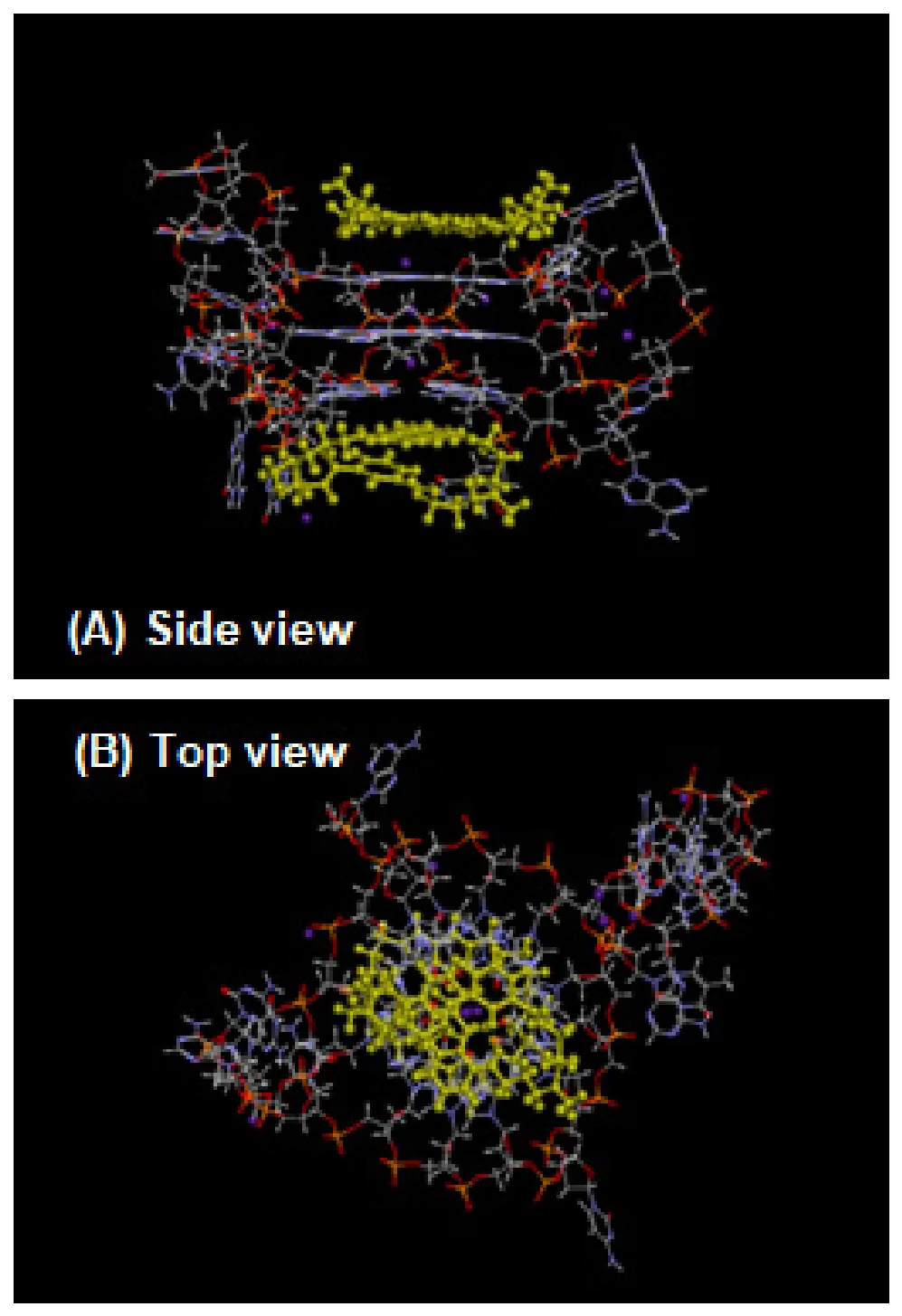
AlphaFold3-predicted structural model of the cAQ-mBen–mt6363 complex. Two cAQ-mBen molecules were included in the modeling input based on the apparent 2:1 binding model obtained from UV–visible titration analysis, and potassium ions were included. cAQ-mBen molecules are shown in yellow, and potassium ions are shown as purple spheres. The resulting model places the ligands near opposite terminal G-quartet faces of mt6363 and suggests possible π–π stacking interactions. The model is presented as a computational hypothesis and does not independently validate the binding stoichiometry or binding pose.

**Figure 6:**
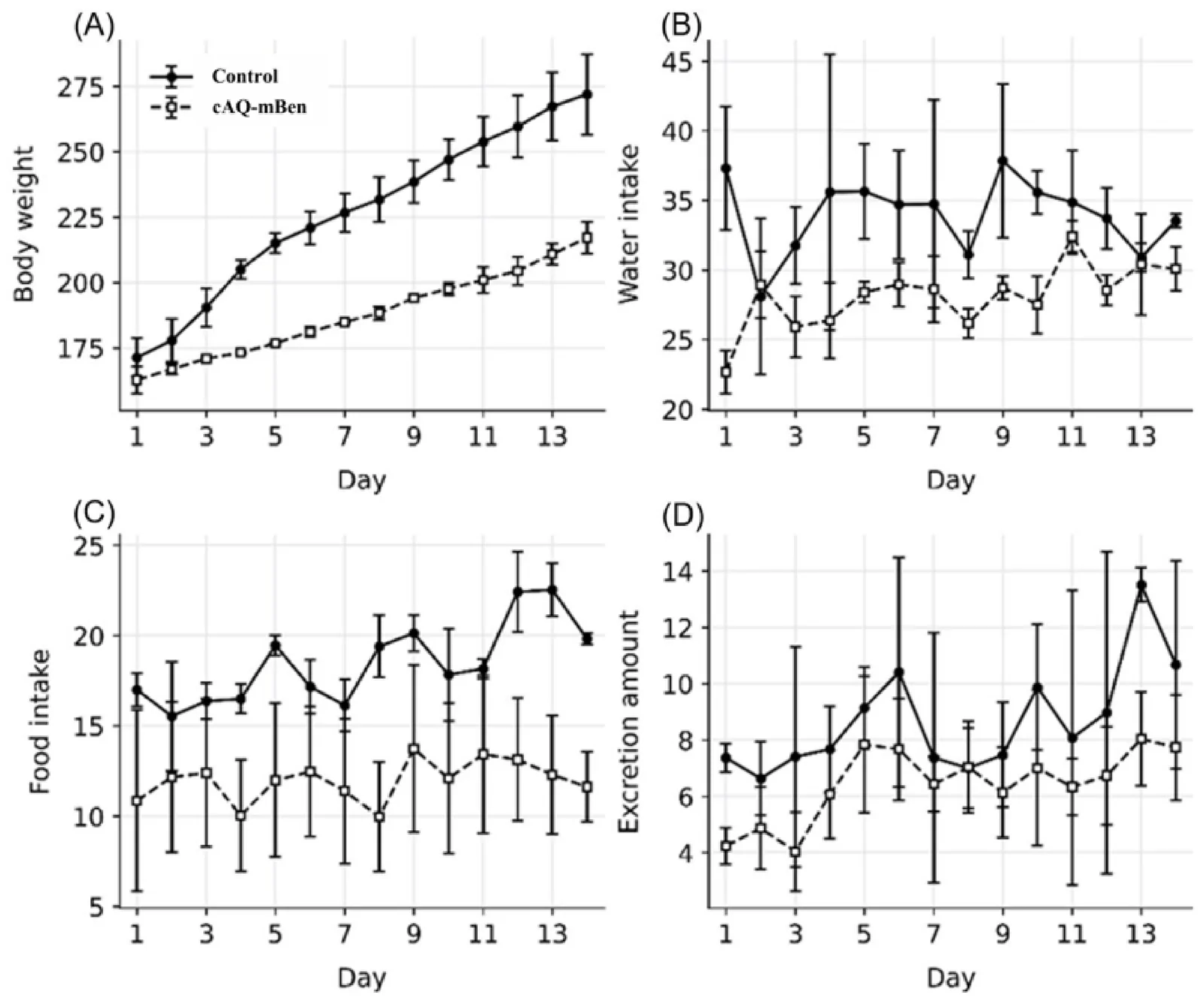
Time-course changes in body weight (**A**), water intake (**B**), food intake (**C**), and excretion amount (**D**) in the control and cAQ-mBen groups. Data are presented as the mean ± SD (*n* = 3 per group). Control, solid line with filled circles; cAQ-mBen, dashed line with open squares.

**Figure 7:**
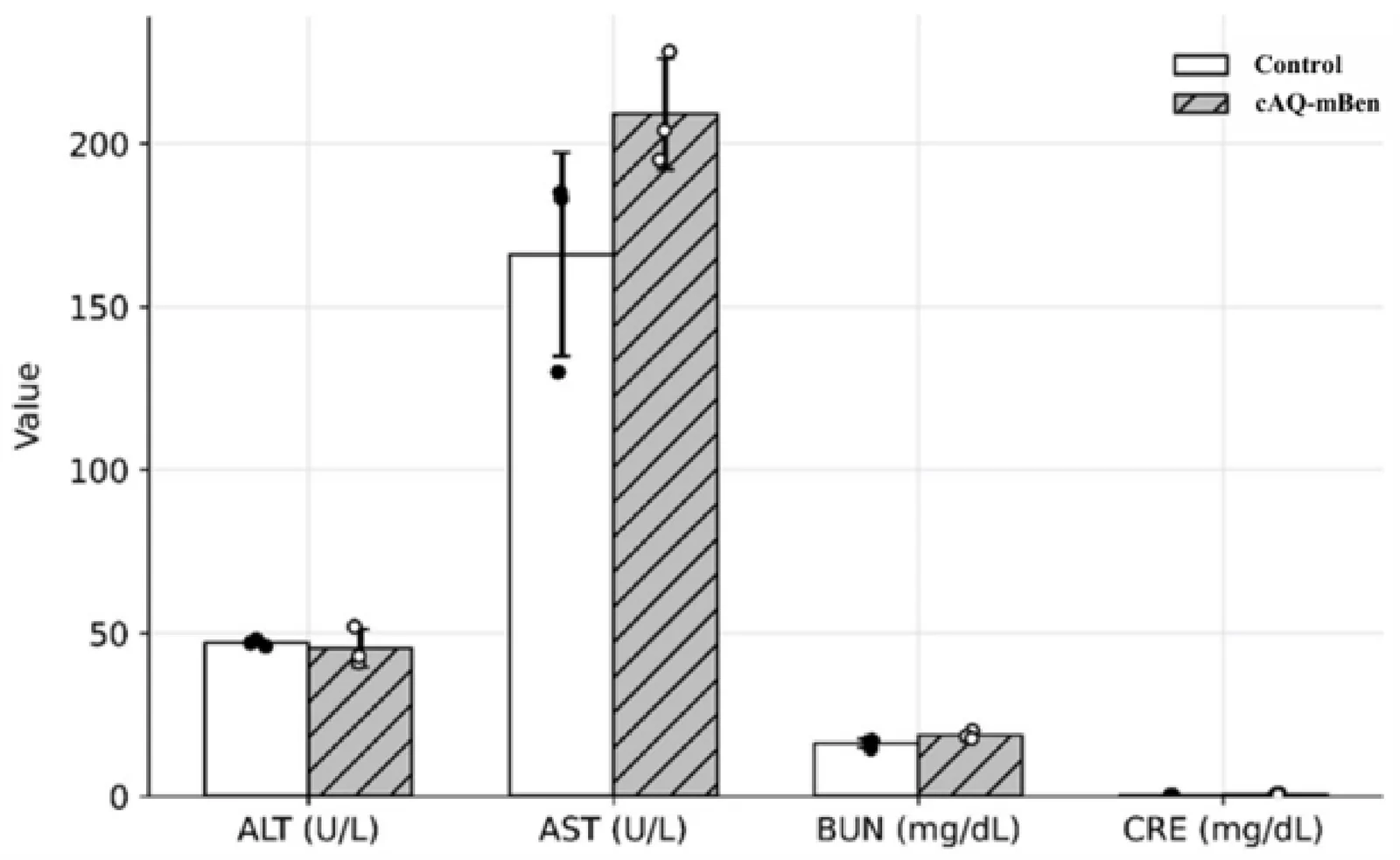
Blood biochemistry in the control and cAQ-mBen groups. Bars indicate the mean ± SD (*n* = 3 per group), and symbols indicate individual animals. Control, white bars with filled circles; cAQ-mBen, gray hatched bars with open circles.

**Figure 8:**
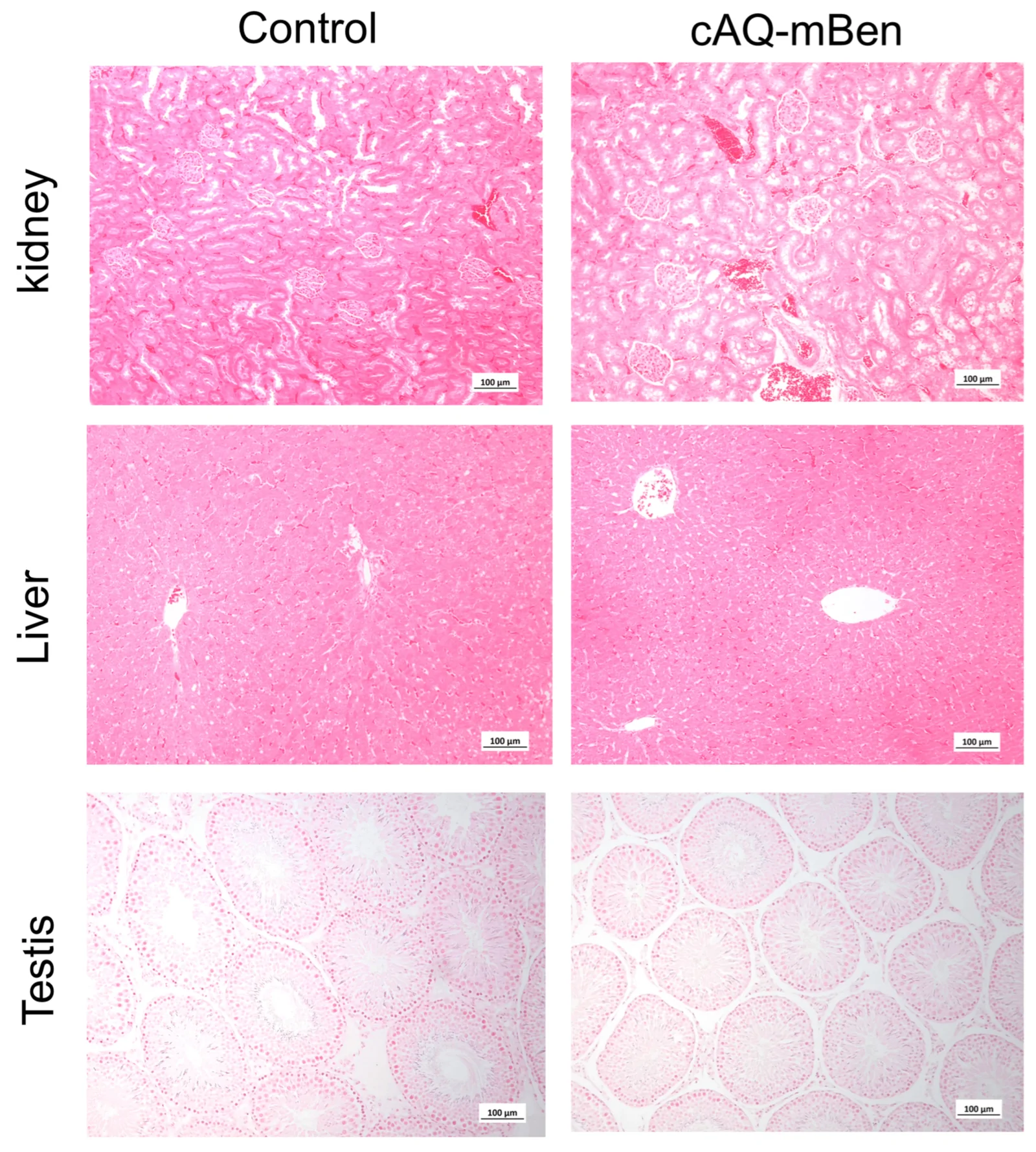
Representative hematoxylin and eosin (H&E)-stained sections of the kidney, liver, and testis from the control and cAQ-mBen-treated rats. The displayed fields are illustrative representative images; histopathological interpretation was based on microscopic examination of the H&E-stained sections by a pathologist. Original magnification: ×100. Scale bar: 100 μm.

## Data Availability

The RNA-Seq dataset analyzed in this study is publicly available in the Gene Expression Omnibus (GEO) under accession number GSE223310. Other data supporting the findings of this study are available within the article and its Supplementary Materials.

## Supplementary Materials

The following supporting information can be downloaded at: https://www.mdpi.com/article/10.3390/molecules31193397/s1. Figure S1. Comparison of fixed 1:1 and 2:1 binding models; Figure S2. Residual analysis of the fixed 1:1 and 2:1 binding models; Figure S3. Mole-ratio analysis of the cAQ-mBen–mt6363 interaction; Figure S4. Unnormalized CD melting profiles of mt6363; Figure S5. Effects of cAQ-mBen treatment on mitochondrial gene expression; Table S1. Goodness-of-fit parameters for fixed 1:1 and 2:1 binding models; Table S2. Differential expression of mitochondrial DNA-encoded protein-coding genes following cAQ-mBen treatment.
