# Supplementary material for: In Vitro Binding to a Mitochondrial G-Quadruplex-Forming Sequence and Exploratory Acute Tolerability Assessment of a Cyclic Anthraquinone Anticancer Agent
Source: Molecules. 2026 Sep 24;31(19):3397. doi: 10.3390/molecules31193397 (PMC13666387; doi:10.3390/molecules31193397)
Supplement: Supplementary file 1 [file molecules-31-03397-s001.zip › molecules-4547476-supplementary.pdf]

Supplementary Materials

In Vitro Binding to a Mitochondrial G-Quadruplex-Forming Sequence and Exploratory Acute Tolerability Assessment of a Cyclic Anthraquinone Anticancer Agent

Hikaru Fukuda [1], Yukina Sanada [2], Satoshi Fujii [3], Kazuhisa Fujimoto [4], Masaki Morishita [5], Naomi Yada [6], Osamu Takahashi [1], Manabu Habu [1], Shigeori Takenaka [2], Hiroshi Takeuchi [7,*] and Shinobu Sato [2,*]

1. Department of Science of Physical Functions, Division of Maxillofacial Surgery, Kyushu Dental University, 2-6-1 Manazuru, Kokurakita-ku, Kitakyushu 803-8580, Fukuoka, Japan; r07takahashi@fa.kyu-dent.ac.jp (O.T.)
2. Department of Applied Chemistry, Kyushu Institute of Technology, 1-1 Sensui-cho, Tobata-ku, Kitakyushu 804-8550, Fukuoka, Japan
3. Department of Bioscience and Bioinformatics, Kyushu Institute of Technology, 680-4 Kawazu, Iizuka 820-8502, Fukuoka, Japan; sfujii@bio.kyutech.ac.jp
4. Department of Applied Chemistry and Biochemistry, Kyushu Sangyo University, 2-3-1 Matsukadai, Higashi-ku, Fukuoka 813-8503, Fukuoka, Japan
5. Department of Comprehensive Dental Practice Education Development, Kyushu Dental University, 2-6-1 Manazuru, Kokurakita-ku, Kitakyushu 803-8580, Fukuoka, Japan
6. Department of Health Promotion, Division of Oral Pathology, Kyushu Dental University, 2-6-1 Manazuru, Kokurakita-ku, Kitakyushu 803-8580, Fukuoka, Japan; r12yada@fa.kyu-dent.ac.jp
7. Department of Health Promotion, Division of Applied Pharmacology, Kyushu Dental University, 2-6-1 Manazuru, Kokurakita-ku, Kitakyushu 803-8580, Fukuoka, Japan

* Correspondence: r12takeuchi@fa.kyu-dent.ac.jp (H.T.); shinobu@che.kyutech.ac.jp (S.S.)

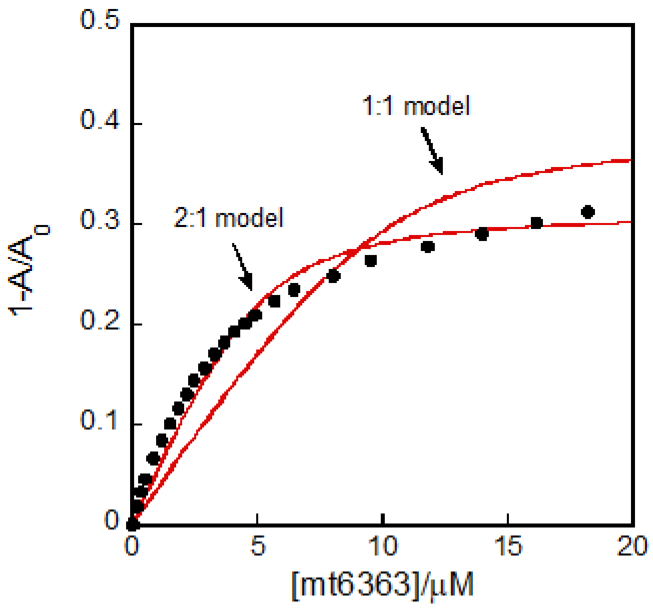

Figure S1. Comparison of fixed 1:1 and 2:1 binding models for the interaction between cAQ-mBen and mt6363. The normalized absorbance change at 533 nm, expressed as $1 - A/A_0$, was plotted as a function of mt6363 concentration at a fixed cAQ-mBen concentration of 10 μM. The experimental data are shown as filled circles. The data were fitted using fixed 1:1 and 2: 1 ligand/DNA binding models. The 2:1 model provided a better fit to the experimental data than the 1:1 model.

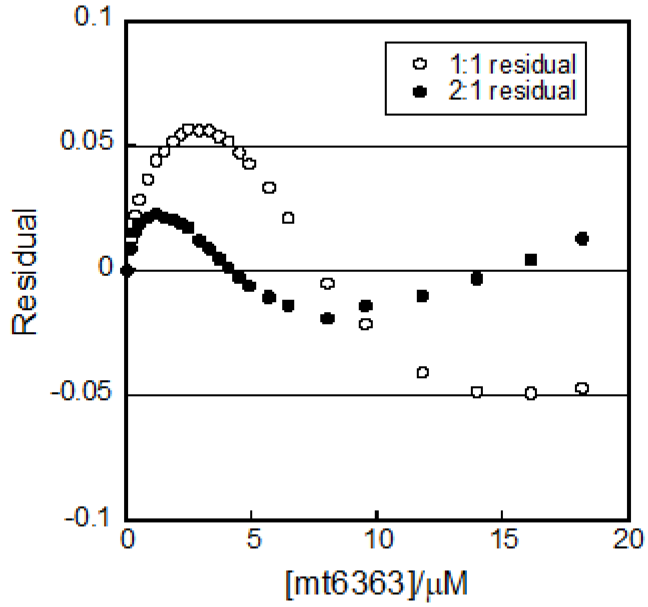

Figure S2. Residual analysis of the fixed 1:1 and 2:1 binding models for the interaction between cAQ-mBen and mt6363. Residuals were calculated as the difference between the experimental and fitted values of the normalized absorbance change at 533 nm. Compared with the 1:1 model, the 2:1 model showed smaller residuals with less systematic deviation over the mt6363 concentration range, supporting that the 2:1 model provides a better description of the experimental data.

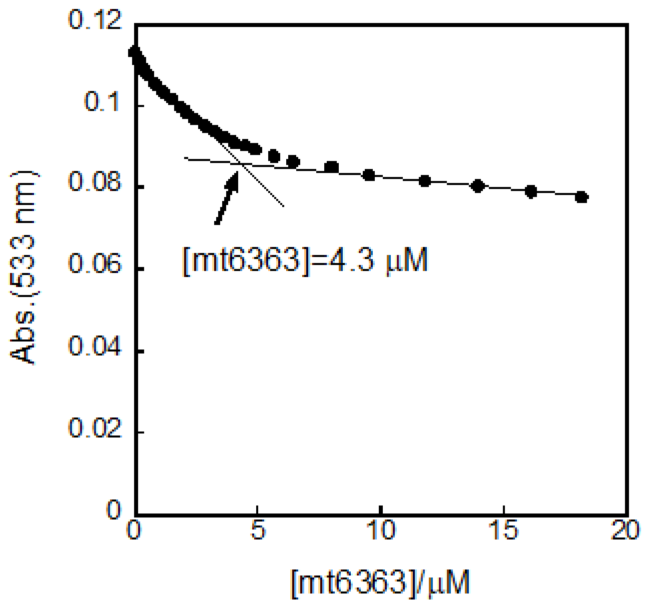

Figure S3. Mole-ratio analysis of the interaction between cAQ-mBen and mt6363. Absorbance at 533 nm was plotted against the mt6363 concentration while the cAQ-mBen concentration was maintained at 10 μM. The breakpoint was determined as the intersection of linear regressions fitted separately to the low- and high-mt6363-concentration regions. The intersection occurred at approximately 4.3 μM mt6363, corresponding to an apparent cAQ-mBen/mt6363 molar ratio of approximately 2.3:1.

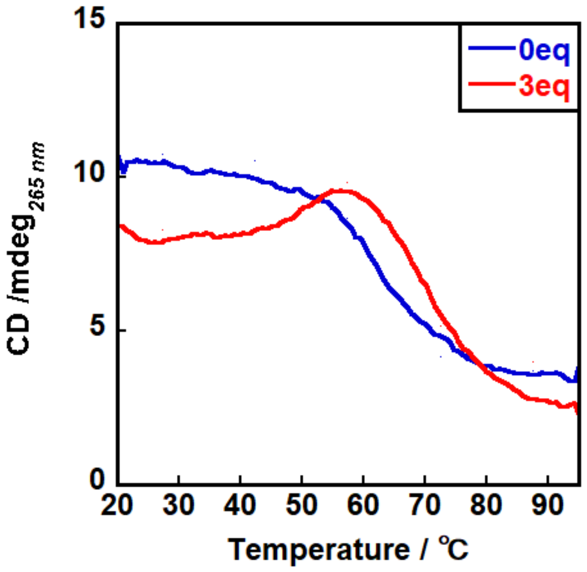

Figure S4. Unnormalized CD melting profiles of mt6363 in the absence and presence of cAQ-mBen. Raw CD signals at 265 nm were monitored as a function of temperature for mt6363 alone and for mt6363 in the presence of a three-fold molar excess of cAQ-mBen. The melting temperature (*T*m) for each condition was determined using the midpoint method.

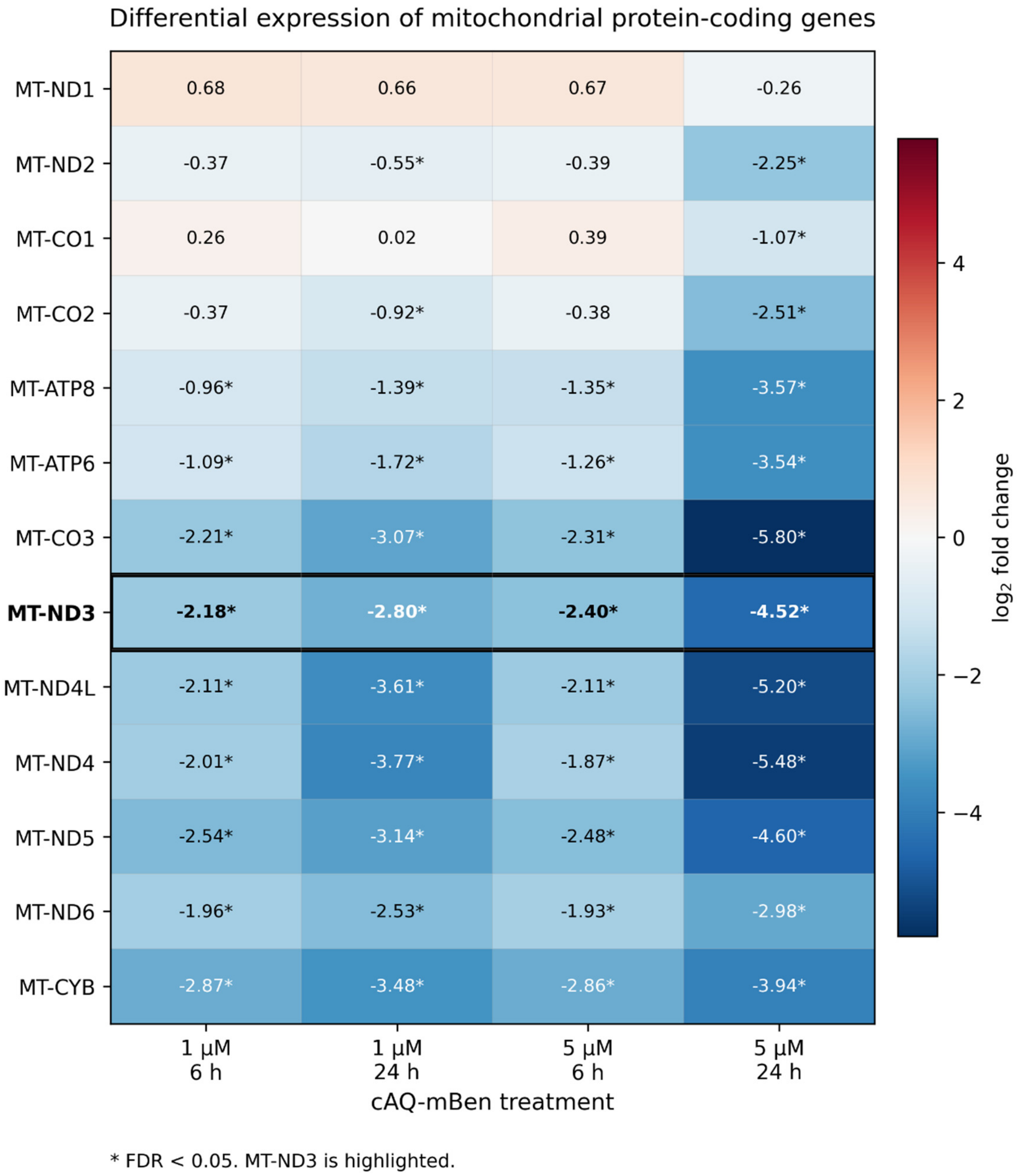

Fig. S5. **Effects of cAQ-mBen treatment on mitochondrial gene expression.** Heatmap showing differential expression of mitochondrial DNA-encoded genes following cAQ-mBen treatment. *MT-ND3*, which contains the mt6363 G-quadruplex-forming sequence, is highlighted.

Table S1. Goodness-of-fit parameters for fixed 1:1 and 2:1 binding models of the cAQ-mBen–mt6363 interaction.

Binding model	R^2	Adjusted R^2	RSS	AIC	BIC	Apparent K_a (M^{-1})	SE of K_a (M^{-1})	95% CI for K_a (M^{-1})
Fixed 1:1	0.7906	0.7707	0.04233	−148.17	−145.81	—*	—*	—*
Fixed 2:1	0.9770	0.9748	0.004658	−201.13	−198.78	7.49×10^5	2.28×10^5	4.60×10^5–2.03×10^6

Note: UV–visible titration data were analyzed using fixed 1:1 and 2:1 binding models. Higher R^2 and adjusted R^2 values and lower RSS, AIC, and BIC values indicate better agreement between the model and the experimental data. The fixed 2:1 model provided a substantially better fit than the fixed 1:1 model. The standard error (SE) of the apparent association constant (K_a) was calculated by error propagation from the fitted K_d value.

*For the fixed 1:1 model, the association constant could not be estimated with a reliable confidence interval under the present fitting conditions.

Table S2. Differential expression of mitochondrial DNA-encoded protein-coding genes following cAQ-mBen treatment

	1 μM, 6 h		1 μM, 24 h		5 μM, 6 h		5 μM, 24 h	
Gene	log2FC	FDR	log2FC	FDR	log2FC	FDR	log2FC	FDR
MT-ND1	0.68	2.39E-01	0.66	1.61E-01	0.67	1.09E-01	-0.26	4.98E-01
MT-ND2	-0.37	2.78E-01	-0.55	4.98E-02	-0.39	1.13E-01	-2.25	1.27E-06
**MT-ND3**	**-2.18**	**1.27E-06**	**-2.80**	**4.12E-08**	**-2.40**	**1.47E-07**	**-4.52**	**1.99E-10**
MT-ND4	-2.01	5.54E-03	-3.77	1.31E-04	-1.87	1.73E-03	-5.48	3.20E-06
MT-ND4L	-2.11	5.77E-06	-3.61	2.40E-08	-2.11	1.56E-06	-5.20	2.39E-10
MT-ND5	-2.54	2.67E-04	-3.14	4.43E-05	-2.48	6.94E-05	-4.60	7.05E-07
MT-ND6	-1.96	1.11E-04	-2.53	8.55E-06	-1.93	2.61E-05	-2.98	2.72E-07
MT-CO1	0.26	6.62E-01	0.02	9.67E-01	0.39	2.74E-01	-1.07	6.65E-03
MT-CO2	-0.37	3.50E-01	-0.92	1.05E-02	-0.38	1.61E-01	-2.51	4.05E-06
MT-CO3	-2.21	2.27E-03	-3.07	2.21E-04	-2.31	3.87E-04	-5.80	1.52E-06
MT-ATP6	-1.09	1.16E-02	-1.72	3.76E-04	-1.26	1.14E-03	-3.54	3.97E-07
MT-ATP8	-0.96	2.21E-02	-1.39	1.21E-03	-1.35	4.79E-04	-3.57	4.22E-08
MT-CYB	-2.87	2.03E-05	-3.48	3.72E-06	-2.86	5.35E-06	-3.94	2.12E-07

Notes: SAS cells were treated with cAQ-mBen at 1 or 5 μM for 6 or 24 h. Differential expression was analyzed using edgeR. Genes with FDR < 0.05 were considered significantly differentially expressed. MT-ND3, which contains the mt6363 G-quadruplex-forming sequence, is highlighted.

Source: RNA-seq dataset GEO accession GSE223310.
